# The role of sexual orientation in the relationships between body perception, body weight dissatisfaction, physical comparison, and eating psychopathology in the cisgender population

**DOI:** 10.1007/s40519-020-01047-7

**Published:** 2020-10-22

**Authors:** Paolo Meneguzzo, Enrico Collantoni, Elisa Bonello, Mariantonietta Vergine, Simone C. Behrens, Elena Tenconi, Angela Favaro

**Affiliations:** 1 Department of Neuroscience, University of Padova, Via Giustiniani 2, 35128 Padova, Italy; 2 Padova Neuroscience Center, University of Padova, Padova, Italy; 3 Department of Psychosomatic Medicine and Psychotherapy, Medical University Hospital Tübingen, Tübingen, Germany; 4 Department of Perceiving Systems, Max Planck Institute for Intelligent Systems, Tübingen, Germany

**Keywords:** Body image, Sexual orientation, Gender, Eating disorders, Body weight dissatisfaction

## Abstract

**Purpose:**

Body weight dissatisfaction (BWD) and visual body perception are specific aspects that can influence the own body image, and that can concur with the development or the maintenance of specific psychopathological dimensions of different psychiatric disorders. The sexual orientation is a fundamental but understudied aspect in this field, and, for this reason, the purpose of this study is to improve knowledge about the relationships among BWD, visual body size-perception, and sexual orientation.

**Methods:**

A total of 1033 individuals participated in an online survey. Physical comparison, depression, and self-esteem was evaluated, as well as sexual orientation and the presence of an eating disorder. A Figure Rating Scale was used to assess different valences of body weight, and mediation analyses were performed to investigated specific relationships between psychological aspects.

**Results:**

Bisexual women and gay men reported significantly higher BWD than other groups (*p* < 0.001); instead, higher body misperception was present in gay men (*p* = 0.001). Physical appearance comparison mediated the effect of sexual orientation in both BWD and perceptual distortion. No difference emerged between women with a history of eating disorders and without, as regards the value of body weight attributed to attractiveness, health, and presence on social media.

**Conclusion:**

This study contributes to understanding the relationship between sexual orientations and body image representation and evaluation. Physical appearance comparisons should be considered as critical psychological factors that can improve and affect well-being. The impact on subjects with high levels of eating concerns is also discussed.

**Level of evidence:**

Level III: case–control analytic study.

## Introduction

The way people perceive their bodies is an essential aspect of daily intrapersonal and interpersonal interactions; indeed, a negative body image is associated with eating disorders (ED), adverse sexual experiences, low self-esteem, depression, and anxiety [1–3]. Body image is influenced by cultural factors, interpersonal skills, physical changes over time, personality characteristics, self-esteem, and quality of life [4–7]. Recently, the literature has considered genders and sexual orientations as significant factors that might have a significant effect on one’s body evaluation, but bisexual women and men are often misrepresented in the literature and included with other sexual minorities [8–12].

The presence of a discrepancy between the body’s perception and the ideal body that people struggle to achieve can be defined as body image disturbance [13]. Body image disturbance is related to the body dissatisfaction construct, even if it is less linked to the affective elements and more to the perception feature [14, 15]. However, this construct seems more complicated than just the discrepancy between two body sizes [16]. Different elements could be included in the definition of body image disturbance, starting from body perception [17]. Indeed, the body size estimation accuracy is linked to top/down cognitive mechanisms and cognitive schemas that could bias the recall from memory of one’s own body [18–20]. Another element that could be considered in the body image disturbance is body weight dissatisfaction, which refers only to the body’s weight-related visual appearance, showing the evaluation discrepancy reported by subjects [19]. Studies on these elements are growing in the last decades [21] but results are still preliminary in the sexual minority population.

There is a broad consensus that heterosexual women experience normative pressure to achieve specific ideal body sizes [22]. However, lesbian and bisexual women remain underrepresented in the literature, with unclear results due to opposing evidence and mixed sampling methodologies [22–24]. Data have recently suggested that bisexual women are more likely than women with other sexual orientations to internalize shape/weight overvaluation and body dissatisfaction [25]. However, more research is needed to confirm these points. The data in the literature seem to be more consistent when it comes to the higher prevalence of body dissatisfaction in gay than in heterosexual men, which is probably due to the emphasis placed on physical appearance in the gay culture and because of pressure from social and interpersonal factors [9, 26–28]. For example, it has been suggested that—due to different beauty ideals and their minority status—gay men are slightly more vulnerable to body dissatisfaction than heterosexual men [29–32].

The evaluation of one’s body image includes the concerns about body weight and shape, the perception thereof, and the ideal body appearance, linked to a specific mood, self-esteem, and cultural environment [33, 34]. Differences in visual body perception have been shown between genders in heterosexual cisgender, with different brain activation linked to their own body and opposite-sex bodies, but no study is available for sexual minorities [35]. The influence of socio-cultural aspects linked to body appearance could bring gay men and women to show a higher misperception of their body, but also for this aspect, few studies are available, and bisexual persons are misrepresented in the existing literature [28]. However, this is a relevant clinical aspect that is crucial in the treatment of body image distortion in eating disorder patients, and more studies are needed in this field [36].

However, in the literature, the psychometric instruments used have not been sufficiently sensitive to differences between subgroups with different socio-cultural pressures about body evaluation, and this could explain the mixed results obtained in the literature [10, 25]. Body dissatisfaction tasks are devised using assumptions about what shape aspects might be relevant for the specific target group (e.g., focusing on muscularity, thinness, or specific body regions). For this reason, body weight dissatisfaction is typically evaluated with Figure Rating Scales (FRS), which are composed of pictures, drawings, or human models displaying a specific weight range and can separate visual distortion components from distorted attitudes [37]. Unfortunately, body weight dissatisfaction among individuals with different sexual orientations is a neglected topic, even though body evaluation plays a well-known role in how people evaluate their quality of life [38, 39].

Our goal is to study different facets of body weight and body image perception in a significant sample of cisgender women and men with different sexual orientations. Recent evidence from the literature has shown psychological differences between cisgender and transgender people regarding body image and body experiences [40, 41]. Moreover, a recent neuroimaging study has suggested that there could be different network connectivity in the gender dysphoria population as regard own body perception [42]. Thus, only cisgender participants’ inclusion allowed to focus on sexual orientations’ role in body weight dissatisfaction and perception with more robust results, advocating for future research about the relationship between cisgender, transgender, and queer population. Our first hypothesis is that heterosexual women and sexual minority men are more likely than sexual minority women and heterosexual men to have high levels of body dissatisfaction or to be influenced more by social norms regarding their bodies. Second, we want to test if there are differences in body image constructs between different sexual orientations by studying a sample of patients with and without ED symptomatology; our aims are to evaluate the clinical impact of sexual orientation in this specific health field. Third, due to the evidence that physical comparison, eating psychopathology, depression, and self-esteem may influence individuals’ body image, different correlation and mediation analyses were performed. However, due to the fewer evidence from literature, the analyses performed have to be considered exploratory.

## Methods

The data were collected with an anonymous online survey (SurveyMonkey.com) completed by Italian speakers, 18 years old or older. The participants were recruited via online invitations through social media (i.e., Italian Facebook groups related to gender, physical activities, and cultural associations linked to civil rights; both open and close groups) and LGBTQ + group mailing lists from the area of the Veneto Region (Italy), through those responsible for managing personal data, without the involvement of researchers. The invitation consisted of a request to complete voluntarily and spontaneously in questionnaires on body image and body experiences to indicate that the questionnaires would be used for research purposes. The online survey was devised in such a way as to prevent multiple responses from the same IP addresses, but the IP addresses were hidden from investigators. The data were collected between February 2019 and October 2019. Each participant provided informed consent before receiving the survey. The research was in accordance with the Declaration of Helsinki, and an ethical evaluation of the study was obtained by the local research ethics committee. The anonymous nature of the data acquired does not require the application of European legislation about privacy. The duration of the survey was about 30 min.

### Sociodemographic characteristics

As suggested by the international literature, the gender of each participant was determined by asking them whether they identify as cisgender, transgender, or queer with forced choices about the gender they had at birth and about their current gender [43]. Sexual orientation was assessed asking about their sexual orientation with four possible answers: heterosexual, bisexual, gay/lesbian, asexual (“Which of the following best describes you? (1) heterosexual; (2) bisexual; (3) gay or lesbian; (4) asexual”) [44]. Lifetime diagnosis of any ED was determined with an item about a current or past diagnosis of any ED. Besides, demographic information was collected.

### Self-esteem

The Rosenberg Self-Esteem Scale (RSES) is a widely-used tool that measures positive and negative feelings about oneself [45]. It is a 10-item Likert scale with four-point scale answers on a continuum ranging from “strongly agree” to “strongly disagree.” The total score indicates how high or low a subject’s self-esteem is. The reliability of the scale in this study was high (Cronbach’s *α* = 0.815).

### Physical comparisons at social events

The Physical Appearance Comparison Scale (PACS) [46] was used to measure the subjects’ tendency to compare their physical appearances with others in various social situations. The Italian version was already validated in a previous study [47]. The PACS is a 5-item Likert scale with five possible answers, ranging from “never” to “always.” The total score indicates the extent of an individual’s appearance-related social comparison tendencies. The reliability of the scale in this study was sufficient (Cronbach’s *α* = 0.752).

### Psychological well-being

The Patient Health Questionnaire-9 (PHQ-9) is a screening tool for depression [48]. It is a 9-item Likert scale, and each item evaluates the presence of one of the DSM criteria for a depressive episode in the prior 2 weeks. There are four answer categories: 0 (“not at all”), 1 (“few days”), 2 (“more than half the days”), and 3 (“almost every day”). The total score is obtained by adding up the scores for each item, and higher scores indicate greater depression symptomatology, with a score between 8 and 11 denoting major depression. The reliability of the scale in this study was sufficient (Cronbach’s *α* = 0.788).

### Eating concerns

The Eating Attitudes Test (EAT-26) is a well-established self-report questionnaire measuring eating symptoms and concerns characteristic of individuals with and without ED [49]. It includes 26 items that are rated on a 6-point Likert scale ranging from 1 (“neve”) to 6 (“always”), and it comprises three subscales (dieting, bulimia and food preoccupation, and oral control), which can be combined to yield a total EAT-26 score. The higher is the score, the higher is the number of ED symptoms present in an individual; a value of 20 points or higher indicates a probable ED diagnosis [50, 51]. The reliability of the scale in this study was sufficient (Cronbach’s *α* = 0.898).

### Figure Rating Scales

Biometric Figure Rating Scales (FRSs) are reliable and efficient tools for assessing body image disturbances as long as the scale is representative of the weight range and centers roughly around the average weight of the target population [33]. Similarly to the procedure described in the literature [33], two different 12-point biometric FRS were obtained from a statistical model of average shape variation [52] (i.e., one scale for men and one for women). These FRSs cover a BMI spectrum from 14 to 36 kg/m^2^ with an increase of 2 kg/m^2^ and a center of 25 kg/m^2^. The participants were asked to choose which figure best represented their body size and represented an ideal body; these questions were asked to identify possible biased representations and body weight dissatisfaction. Body weight dissatisfaction was evaluated by subtracting the selected ideal figure score from the estimated current figure score. Perceptual distortion was assessed by subtracting the current figure score (i.e., the individual’s body’s score, which was closest to self-reported BMI) from the estimated current figure score [33]. Participants were also asked to choose a human model that represented the body shapes they consider to be the healthiest and most attractive. For each question, the participants could choose only one human model from the male FRS and one from the female FRS. This part of the study’s results were obtained by analyzing the body mass indices of the average human model selected. Previous studies have demonstrated the validity of using a visual rating scale to assess different constructs linked to body sizes and weight, such as attractiveness or cognitive representation [53].

### Data analysis

The entire analysis was conducted with IBM SPSS Statistics 23.0 (SPSS, Chicago, IL, USA). Differences between subgroups in terms of body dissatisfaction and the perceived pressure from social norms were explored using MANOVAs and Bonferroni-corrected post hoc tests. To evaluate the clinical impact of sexual orientation on the prevalence and extent of EDs, Chi-square tests, and MANOVAs were used to compare participants with and without ED symptoms. Since the groups differed significantly in age, we conducted analyses of covariance by applying general linear models (GLMs) with age and BMI as covariates. Pearson correlations between variables were calculated to evaluate the contribution of other variables to the relationship between sexual orientation and body weight dissatisfaction.

Several mediation analyses were performed with the SPSS PROCESS vers. 3.4 macro-extension [54], applying Model 4. We used eating concerns, social physical comparison, depression, and self-esteem as possible mediators, to determine if these psychological constructs can mediate the relationship between sexual orientation (independent variable) and body weight dissatisfaction (dependent variable). This model was chosen in order to evaluate each construct independently as a mediator. According to the recent literature, sexual orientation was considered a continuous variable from heterosexual orientation to gay/lesbian, but it has been collapsed into a discrete variable for statistical analysis purposes [55–57]. This procedure estimates the total, direct, and indirect effects of multiple predictors on a dependent variable via the mediator while controlling for covariates. The PROCESS SPSS macro procedure was selected because of its better performance and statistical power than other mediation approaches (such as the Sobel test) [58]. The bootstrapping sampling distributions of the indirect effects were set to 5000, and they were produced by selecting a sample of cases from the complete data set and calculating the indirect effects in the resamples. Point estimates and confidence intervals were estimated for the indirect effects, and the bias level was set to 95%. The bootstrapping method enables to compare the relative strength of the single indirect effects involved in the multiple mediation model, estimating the statistical significance of the point estimate for the difference between mediators. For this specific study, the procedure showed the direct effect of the independent categorical variable (sexual orientation) on the dependent variable, and the indirect effect through variables. The alpha was set at *p* < 0.05 for all analyses.

## Results

### Sociodemographic and anthropometric characteristics

A total of 1389 subjects provided informed consent and participated in the survey, and 1112 participants completed the survey (80.1%). Furthermore, 1065 subjects (96%) identified themselves as cisgender and were thus included in the analysis. Data from participants who did not complete the survey were excluded from the study, and none of the participants provided gender-mixed answers. Only 12 women identified themselves as asexual and were excluded from the analysis as these individuals’ sample was too small. Moreover, no men identified as asexual. Only five men reported a lifetime diagnosis of an ED and were excluded from the analysis because of the small number of individuals. The demographic characteristics of the 375 men and 574 women without a lifetime diagnosis of any ED are presented in Table 1. The sexual orientation groups differed in terms of age, BMI, and education levels; however, BMI differences across sexual orientation groups were only present in the female samples.

**Table 1:** Cisgender participant characteristics

	Women (*N* = 574)						Men (*N* = 378)					
	HEW, *N* = 465	BIW, *N* = 78	Lesbian, *N* = 31	*F*	*p*	Post hoc (*p*)	HEM, *N* = 272	BIM, *N* = 50	Gay, *N* = 56	*F*	*p*	Post hoc (*p*)
Age, years	28.05 (8.04)	24.99 (4.17)	24.68 (3.99)	7.904	**< 0.001**	HEW > BIW (0.003) HEW > Lesbian (0.045)	28.79 (7.65)	26.04 (5.29)	29.82 (7.52)	3.876	**0.022**	HEM > BIM (0.048) Gay > BIM (0.026)
BMI, kg/m^2^	22.44 (6.82)	24.47 (6.16)	23.18 (5.20)	1.778	0.102		23.64 (3.66)	22.34 (2.27)	23.93 (3.14)	1.551	0.090	
BMI min, kg/m^2^	19.64 (5.61)	21.40 (5.53)	20.54 (3.64)	3.603	**0.028**	BIW > HEW (0.028)	21.52 (3.53)	20.58 (1.81)	21.20 (2.61)	1.841	0.160	
BMI max, kg/m^2^	24.57 (7.86)	27.00 (7.04)	24.90 (5.21)	3.400	**0.034**	HEW > BIW (0.028)	25.77 (3.53)	23.98 (3.97)	26.72 (4.35)	2.677	0.070	
Ethnicity % caucasian	97.63	100.00	96.77	*χ*^2^ = 5.324	0.868		97.79	100.00	100.00	*χ*^2^ = 2.376	0.305	

Descriptive characteristics of the investigated subgroups. Values are means (standard deviation), if not otherwise specified. Only significant post hoc contrast is reported

*HEW* heterosexual women, *BIW* bisexual women, *HEM* heterosexual men, *BIM* bisexual men, *BMI* body mass index, *F* ANOVA with Bonferroni-corrected post hoc tests, as well as Chi-square tests, were conducted for the group demographic characteristics

No difference was found for the marriage status; single women: 72.3% of heterosexual, 74.3% of bisexual, 83.9% of lesbian (*χ*^2^ = 4.214, *p* = 0.378 for women); single men: 80.15 of heterosexual, 88.0% of bisexual, 82.14% of gay men (*χ*^2^ = 2.873, *p* = 0.579 for men).

### Differences in body comparison, self-esteem, general health and eating concerns

The following abbreviations were used for sexual orientation subgroups: HEW (heterosexual women), BIW (bisexual women), HEM (heterosexual men), and BIM (bisexual men). Table 2 presents the means, standard deviations, and other statistics illustrating group differences. Gay men (5.3% of the sample) and BIW (7.3% of the sample) were more likely than their heterosexual peers to demonstrate eating concerns. The PACS scores indicate that gay men and HEM were less likely than their peers to engage in physical comparison, with significant differences between sexual orientations. Furthermore, gay men and BIW had lower self-esteem than HEM and HEW, and BIW had higher depression levels than HEW and gay men.

**Table 2:** Psychological evaluation of cisgender responders

	Women						Men					
	HEW	BIW	Lesbian	*F*	*p* *η*_*p*_^2^	Post hoc (*p*)	HEM	BIM	Gay	*F*	*p* *η*_*p*_^2^	Post hoc (*p*)
RSES	16.92 (4.90)	15.27 (5.12)	16.71 (4.17)	3.797	**0.023** 0.026	HEW > BIW (0.018)	17.96 (4.64)	16.80 (3.93)	16.25 (4.12)	4.219	**0.015** 0.022	HEM > Gay (0.028)
PACS	14.60 (3.82)	13.90 (3.68)	11.55 (3.05)	10.196	**< 0.001** 0.016	BIW > Lesbian (0.010) HEW > Lesbian (< 0.001)	12.51 (3.62)	16.00 (2.84)	15.50 (3.26)	33.210	**< 0.001** 0.150	BIM > HEM (< 0.001) Gay > HEM (< 0.001)
PHQ-9	8.14 (4.57)	10.44 (4.94)	8.81 (4.87)	8.263	**< 0.001** 0.033	BIW > HEW (0.000)	7.03 (4.51)	8.12 (4.60)	6.89 (3.93)	1.386	0.251 0.007	
EAT26-TOTAL	6.33 (4.64)	7.28 (4.87)	6.21 (4.63)	1.478	0.229 0.018		5.38 (4.98)	4.72 (3.92)	7.46 (4.85)	5.270	**0.006** 0.027	Gay > HEM (0.011) Gay > BIM (0.011)
FRS PD	− 0.48 (1.34)	− 0.65 (1.45)	− 0.76 (1.18)	0.964	0.382 0.001		− 0.36 (1.26)	− 0.60 (1.64)	0.32 (1.83)	6.697	**0.001** 0.035	Gay > HEM (0.004) Gay > BIM (0.003)
FRS BWD	1.96 (1.94)	3.05 (2.41)	1.61 (1.78)	10.852	**< 0.001** 0.014	BIW > HEW (< 0.001) BIW > Lesbian (0.002)	1.00 (1.79)	0.36 (1.40)	2.07 (2.38)	12.158	**< 0.001** 0.061	Gay > HEM (< 0.001) Gay > BIM (< 0.001)
Attractive female body	16.93 (2.11)	18.92 (3.49)	17.81 (1.92)	24.532	**< 0.001** 0.079	BIW > HEW (< 0.001)	18.06 (1.99)	20.00 (4.52)	17.85 (2.33)	13.063	**< 0.001** 0.067	BIM > HEM (< 0.001) BIM > Gay (< 0.001)
Healthy female body	17.81 (2.37)	18.54 (1.85)	17.75 (1.50)	3.351	**0.036** 0.012	BIW > HEW (< 0.001)	18.69 (2.14)	17.75 (2.13)	19.26 (1.99)	6.608	**0.002** 0.035	Gay > BIM (0.001) HEM > BIM (0.015)
Attractive male body	21.04 (2.06)	21.95 (2.90)	20.56 (2.17)	6.531	**0.002** 0.022	BIW > HEW (0.003) BIW > Lesbian (0.009)	20.39 (2.18)	21.25 (4.28)	20.67 (2.85)	3.218	**0.040** 0.012	BIM > HEM (0.041)
Healthy male body	20.91 (2.07)	20.92 (1.52)	20.44 (1.99)	0.865	0.422 0.003		20.60 (2.42)	20.00 (1.01)	21.19 (2.65)	3.308	**0.038** 0.018	Gay > BIM (0.032)

Means (standard deviations) of the psychological variables per gender and sexual orientation. For attractiveness and health representation the table reports the BMI of the human model selected in the FRS

*RSES* Rosenberg Self-Esteem Scale, *PACS* Physical Appearance Comparison Scale, *PHQ-9* Patient Health Questionnaire-9, *EAT26* Eating Attitudes Test, *HEW* heterosexual women, *BIW* bisexual women, *HEM* heterosexual men, *BIM* bisexual men, *BMI* body mass index, *FRS* Figure Rating Scale, *PD* perceptual distortion, *BWD* body weight dissatisfaction, *η*_*p*_^*2*^ partial eta squared, *F* ANOVA with Bonferroni-corrected post hoc tests, only significant post hoc contrast are reported

Different ANOVA analyses were performed both for the FRS PD (*F* (999, 5) = 3.259, *p* = 0.006, *η*_*p*_^2^ = 0.016) and the FRS BWD (*F* (1027, 5) = 29.002, *p* < 0.001, *η*_*p*_^2^ = 0.124), considering all the subgroups together. Table 2 shows the post hoc comparison between the women and men subgroups. On the other hand, as regards the FRS PD results, the post hoc analyses between the two different genders showed a significant difference only between HEW and gay men (*p* = 0.002). As regards the FRS BWD, HEW showed significantly higher dissatisfaction than HEM (*p* < 0.001) and BIM (*p* < 0.001, *p* < 0.001); BIW showed higher dissatisfaction than HEM (*p* < 0.001), BIM (*p* < 0.001), and gay men (*p* = 0.001); and lesbian women showed higher body weight dissatisfaction than BIM (*p* = 0.002).

Significant differences were also confirmed with the GLM analyses using age and BMI as covariates, which demonstrates that age and BMI do not affect the differences mentioned above.

The respondents’ visual perception, body weight dissatisfaction, and specific mentalized body representations are summarized in Table 2. Women did not exhibit differences in perceptual distortions and chose human models that are close to their actual BMIs; gay men, however, significantly overestimated their body sizes. Body weight dissatisfaction was higher in BIW and gay men than their peers.

**Fig. 1:**
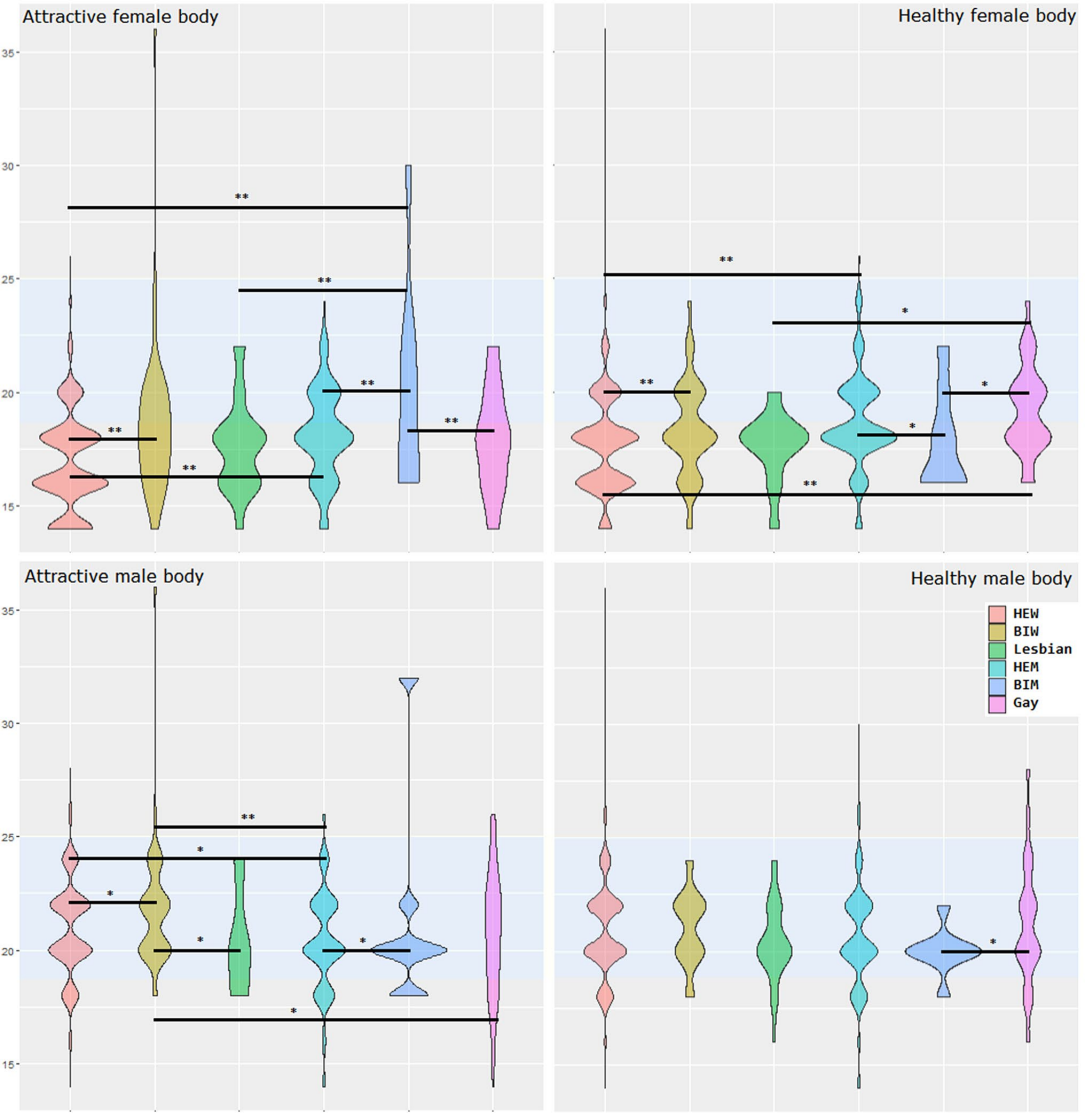
Attractive body shape selection. This figure is the graphic representation of data from Table 2. For each sexual orientation group, the violin plot showed the distribution of the FRS responses for attractive and healthy female and male bodies. The light blue area showed a normal BMI (> 18.5 kg/m^2^ and < 25.0 kg/m^2^). Significant differences are showed with continuous lines between subgroups. From the figure, it is possible to appreciate how most of the female bodies' responses are in the underweight area, which does not happen in males. Moreover, the different distributions of the responses could be appreciated thanks to the graphs’ shapes, even without different means. *p < 0.05; **p < 0.001

As regards the mentalized ideal body weight, BIW were more likely than HEW to find male and female bodies with higher BMIs more attractive, and BIM were more likely than HEM to find these bodies more attractive. Figure 1 provides a graphic representation of the participants’ views on the attractiveness of body shapes. No differences were found in the GLM analyses with age and BMI as covariates. Comparisons between sexual orientation subgroups without gender separation: gay men are the subgroup with the highest perceptual distortion, which was calculated based on individuals' ability to choose their corresponding human model (*p* = 0.006). Regarding body weight dissatisfaction, BIW had the highest average among all subgroups, but it is not different from the scores for lesbians and gay men.

Different correlation analyses have been performed between variables, showing different relationships between dependent constructs in different sexual orientations. See Table 3 for the data.

**Table 3:** Pearson’s correlation between psychological characteristics and figurate rating scale scores

	1)	2)	3)	4)	5)	6)	7)	8)
HEW								
1) Age	–							
2) BMI	0.133**	–						
3) RSES	0.156**	− 0.038	–					
4) PACS	− 0.074	0.015	− 0.407**	–				
5) PHQ9	− 0.132**	0.024	− 0.612**	0.309**	–			
6) EAT26-TOTAL	− 0.148**	0.028	− 0.328**	0.278**	0.389**	–		
7) FRS PD	0.135**	− 0.112*	− 0.187**	0.218**	0.129**	0.069	–	
8) FRS BWD	0.203**	0.418**	− 0.219**	0.268**	0.212**	0.214**	0.569**	–
BIW								
1) Age	–							
2) BMI	− 00.029	–						
3) RSES	0.110	− 0.202	–					
4) PACS	− 0.119	0.335**	− 0.521**	–				
5) PHQ9	0.059	0.192	− 0.700**	0.244*	–			
6) EAT26-TOTAL	0.016	0.349**	− 0.348**	0.433**	0.323**	–		
7) FRS PD	− 0.146	− 0.100	− 0.270*	0.420**	0.131	0.407**	–	
8) FRS BWD	− 0.052	0.444**	− 0.244*	0.483**	0.274*	0.538**	0.268*	–
Lesbian								
1) Age	–							
2) BMI	0.704**	–						
3) RSES	0.107	− 0.067	–					
4) PACS	0.348	0.255	− 0.141	–				
5) PHQ9	0.088	0.349	− 0.584**	− 0.004	–			
6) EAT26-TOTAL	0.145	− 0.044	− 0.595**	0.048	0.503**	–		
7) FRS PD	0.199	0.519**	0.014	0.174	0.228	− 0.188	–	
8) FRS BWD	0.575**	0.877**	− 0.056	0.236	0.379*	− 0.070	0.785**	–
HEM								
1) Age	–							
2) BMI	0.392**	–						
3) RSES	0.038	− 0.017	–					
4) PACS	0.022	− 0.001	− 0.188**	–				
5) PHQ9	− 0.094	0.034	− 0.699**	0.140*	–			
6) EAT26-TOTAL	0.077	0.476**	− 0.204**	0.153*	0.224**	–		
7) FRS PD	0.287**	0.258**	− 0.246**	0.307*	0.214**	0.141*	–	
8) FRS BWD	0.301**	0.770**	− 0.209**	0.094	0.204**	0.447**	0.560**	–
BIM								
1) Age	–							
2) BMI	0.317*	–						
3) RSES	0.574**	0.042	–					
4) PACS	− 0.701**	− 0.624**	− 0.406**	–				
5) PHQ9	− 0.499**	0.044	− 0.669**	0.037	–			
6) EAT26-TOTAL	− 0.562**	− 0.393**	− 0.555**	0.516**	0.687**	–		
7) FRS PD	− 0.787**	− 0.471**	− 0.342**	0.630**	0.042	0.183	–	
8) FRS BWD	− 0.400**	0.348*	− 0.322*	0.278	− 0.045	− 0.093	0.256	–
Gay								
1) Age	–							
2) BMI	0.054	–						
3) RSES	0.177	− 0.012	–					
4) PACS	0.124	− 0.104	− 0.185	–				
5) PHQ9	− 0.274*	− 0.077	− 0.533**	0.183	–			
6) EAT26-TOTAL	0.128	0.095	− 0.370**	0.362**	0.445**	–		
7) FRS PD	− 0.167	0.025	0.037	0.387**	0.253	0.188	–	
8) FRS BWD	0.007	0.606**	0.080	0.173	0.114	0.287*	0.604**	–

Pearson correlations between the assessed psychological variables

*RSES* Rosenberg Self-Esteem Scale, *PACS* Physical Appearance Comparison Scale, *PHQ-9* Patient Health Questionnaire-9, *EAT26* Eating Attitudes Test, *HEW* heterosexual wsomen, *BIW* bisexual women, *HEM* heterosexual men, *BIM* bisexual men, *FRS* Figure Rating Scale, *PD* perceptual distortion, *BWD* body weight dissatisfaction

*Correlation is significant at the 0.05 level (two-tails); **correlation is significant at the 0.01 level (two-tails)

### Comparisons based on lifetime diagnosis of EDs

Eighty-one women reported a lifetime diagnosis of an ED and scored higher than the group without an ED on all the psychological evaluation scales. There were no significant differences between the demographic profile of this group and that of other women. Still, significant differences between the psychopathological profiles were found; women with an ED diagnosis in all the subgroups had significantly higher scores for PHQ-9, EAT26, and PACS and lower self-esteem scores. Regarding perceptual distortion, women with an ED diagnosis did not show any significant difference in the subgroup analysis, but they had a different profile than women without an ED. Indeed, in the HEW samples, women with an ED had a lower own-body misperception (i.e., they were more accurate in the selection of their human model). However, sexual minority women with an ED were more likely to exhibit perceptual distortions than sexual minority women without an ED. All the subgroups with an ED were more dissatisfied than women without an ED. In terms of the selection of healthy bodies, there were few differences between women with and without an ED; only the attractive female body had a significantly higher BMI in HEW and BIW without ED. See Table 4 for more details.

**Table 4:** Comparison between women with an eating disorder and without an eating disorder

	Women with ED					Women with ED vs. without ED		
	HEW *N* = 54	BIW *N* = 22	Lesbian *N* = 5	*F*	*p*	HEW *t* (*p*)	BIW *t* (*p*)	Lesbian *t* (*p*)
Age, years	27.22 (7.22)	25.41 (4.58)	24.80 (6.57)	0.787	0.459	0.526 (0.469)	0.168 (0.682)	0.003 (0.954)
BMI, kg/m^2^	23.08 (5.66)	23.95 (4.49)	23.14 (1.99)	0.219	0.804	0.442 (0.507)	0.137 (0.712)	0.000 (0.985)
BMI min, kg/m^2^	19.30 (4.42)	18.92 (3.87)	22.04 (2.40)	1.146	0.323	0.174 (0.677)	3.856 (0.052)	0.788 (0.381)
BMI max, kg/m^2^	25.93 (5.61)	27.59 (4.75)	34.06 (13.13)	4.377	**0.016**	1.531 (0.217)	0.136 (0.713)	8.162 (**0.007**) ED > N-ED
RSES	11.29 (4.43)	9.50 (2.11)	9.40 (6.06)	1.808	0.171	65.025 (**< 0.001**) N-ED > ED	26.492 (**0.000**) N-ED > ED	11.681 (**0.002**) N-ED > ED
PACS	17.61 (3.58)	17.27 (5.81)	19.80 (1.64)	0.733	0.484	30.322 (**0.000**) ED > N-ED	10.926 (**0.001**) ED > N-ED	34.314 (**< 0.001**) ED > N-ED
PHQ-9	13.85 (4.27)	14.14 (4.00)	12.00 (6.00)	0.507	0.604	76.583 (**< 0.001**) ED > N-ED	10.383 (**0.002**) ED > N-ED	1.747 (0.195)
EAT26-TOTAL	35.06 (14.20)	33.27 (11.45)	35.40 (18.92)	0.171	0.843	801.712 (**< 0.001**) ED > N-ED	247.918 (**0.000**) ED > N-ED	65.626 (**< 0.001**) ED > N-ED
FRS PD	− 0.04 (1.90)	0.82 (1.56)	0.80 (2.05)	1.918	0.154	4.336 (**0.038**) N-ED > ED	16.801 (**0.000**) ED > N-ED	5.908 (**0.021**) ED > N-ED
FRS BWD	4.22 (2.67)	4.86 (2.23)	4.80 (2.05)	1.724	0.129	15.754 (**< 0.001**) ED > N-ED	10.000 (**0**.**002**) ED > N-ED	13.258 (**0**.**001**) ED > N-ED
Attractive female body	15.81 (2.038)	16.75 (2.29)	15.33 (1.15)	1.354	0.266	− 3.310 (**0.001**) N-ED > ED	− 2.371 (**0.0320**) N-ED > ED	− 2.176 (**0.037**) N-ED > ED
Healthy female body	17.58 (3.42)	17.25 (2.72)	16.00 (0.98)	0.373	0.690	− 0.585 (0.559)	− 1.809 (0.087)	− 1.990 (0.055)
Attractive male body	20.33 (2.54)	21.25 (2.05)	18.00 (0.05)	2.550	0.087	− 2.141 (**0.033**) N-ED > ED	− 0.909 (0.366)	− 6.682 (**< 0.001**) N-ED > ED
Healthy male body	20.84 (3.15)	21.25 (2.05)	20.67 (2.31)	0.133	0.876	− 0.217 (0.879)	0.739 (0.462)	0.213 (0.832)

Descriptives and psychological variables for female participants with eating disorders per sexual orientation and with versus without eating disorders. For mean and SD of women without an eating disorder looked at Table 2. For attractiveness and health, table reports the BMI of the human model selected in the FRS

*HEW* heterosexual women, *BIW* bisexual women, *HEM* heterosexual men, *BIM* bisexual men, *BMI* body mass index, *RSES* Rosenberg Self-Esteem Scale, *PACS* Physical Appearance Comparison Scale, *PHQ-9* Patient Health Questionnaire-9, *EAT26* Eating Attitudes Test, *FRS* Figure Rating Scale, *PD* perceptual distortion, *BWD* body weight dissatisfaction, *ED* eating disorder, *N-ED* no-eating disorder

### Mediation analyses

The first hypothesis tested with mediation analysis was that psychological constructs could mediate the relationship between body weight dissatisfaction and sexual orientation. Looking at women without an ED, the effect of sexual orientation on body weight dissatisfaction was mediated only via the physical appearance comparison (PACS, *β* = − 0.150, SE = 0.044, 95% CI [− 0.244, − 0.072]). Looking at men, the effect of sexual orientation on body weight dissatisfaction was significantly mediated only via eating concerns (EAT26, *β* = 0.102, SE = 0.044, 95% CI [0.028, 0.196]). Looking at women with an ED, no mediation effects were found.

**Fig. 2:**
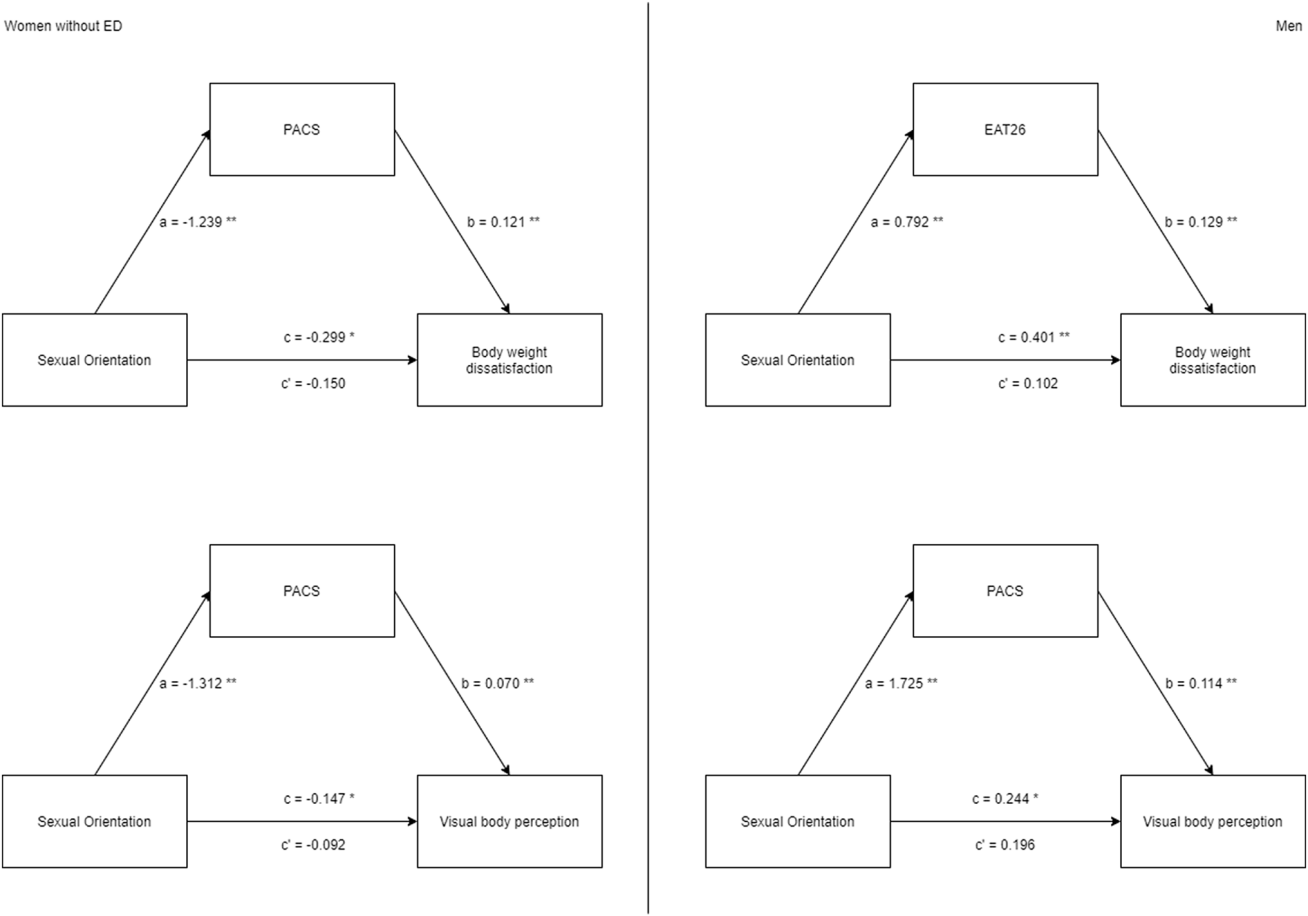
Mediation analysis model. This figure shows the mediation model used. The independent variable used was the sexual orientation, which is a categorical variable and produces mediation results as comparisons between subgroups. The mediators were the psychological data collected: PACS, RSES, PHQ9, and EAT26. Only significant mediation analyses are visualized, *p < 0.05; **p < 0.01

The second hypothesis was about the distorted perception of the body. Women without ED as well as men, showed that the effect of sexual orientation on perceptual body distortion was mediated physical comparison (PACS, women: *β* = − 0.092, SE = 0.030, 95% CI [− 0.158, − 0.039]; men: *β* = 0.196, SE = 0.044, 95% CI [0.116, 0.290]). No significant mediation effect was found in the ED subgroup. See Fig. 2 for details.

## Discussion

The purpose of this study is to provide a comprehensive characterization of body image among cisgender individuals of different sexual orientations. Body image is a crucial aspect of psychological well-being, but the existing literature does not provide a clear picture of the relationship between body image, sexual orientation, and gender. Our data confirmed the literature finding that body image concerns were different across genders and that these concerns have a significant relationship with sexual orientation [59]. Specifically, our data demonstrated that sexual orientation plays a significant role in body weight dissatisfaction in sexual minority groups (especially gay men and BIW) and that different socio-cultural and psychological factors must be considered to understand how people perceive their bodies.

Our primary research goal was to explore the role of gender and sexual orientation in body image. Our first hypothesis was that gay men and HEW were more likely than individuals with other sexual orientations to have body concerns. Our results only partially confirmed our hypothesis, but they are in line with data from the literature that indicate that BIW and gay men are more likely to have body concerns than their heterosexual peers [24, 60, 61]. Indeed, data from recent studies suggest that BIW feel pressured to integrate physical norms from both the heterosexual and lesbian cultures and are significantly vulnerable to body dissatisfaction, even if results are not unique [8, 25]. According to our results, sexual minority men are more prone than the other cisgender groups to internalize social pressure about body evaluation. Depression was significantly correlated with body weight dissatisfaction and other psychological factors, confirming that the link between mental wellness and body image is crucial [39].

This study used a visual approach to identify body weight dissatisfaction and ideal body sizes. As for visual body perception distortions, we found no significant differences between BIW, BIM, HEW, and HEM. Gay men were the only group that overestimated their body sizes; the finding is in accordance with other studies that hypothesized a significant impact of socio-cultural standards in the gay culture with a misperception of their real body shapes [62, 63]. Gay men often exhibit a significant drive to become muscular and fear becoming fat, and this could be the foundation of the misperception of their bodies [64]. This misperception could lead them to overestimate their body sizes, which could also lead them to be more likely to have body dissatisfaction and eating concerns. This result is in accordance with the literature suggesting that there is distorted body size cognition among gay men, who exhibit a strong drive to alter their bodies, even when their bodies reflect their ideal body sizes [63]. The literature has demonstrated that among gay men, there is a disconnect between their ideal and actual BMI; these men cognitively misrepresent their body sizes and overestimate their shapes, which leads to unhealthy eating behaviors to achieve a muscular yet thin body [63, 65].

Similarly, according to our results, women with an ED are significantly more likely than other women to overestimate their body size and weight, as reported in the literature [19]. Indeed, this is a core symptom of EDs, and it is also a well-established outcome predictor [66–69]. This similarity between gay men and women with an ED could be interpreted as a result of the struggle of gay men with their body images and how they are exposed to external and internal pressure; these forms of pressure could lead to unhealthy eating behaviors even in the absence of an ED. Moreover, even BIW showed high levels of body weight dissatisfaction. Our findings are in accordance with the literature on the possible conflict between the internalized Western body standards during childhood and the personal acceptance process linked to being part of a non-heterosexual women group [70]. This conflict between two different ideal body shapes could be at the root of the higher vulnerability of BIW to body-related psychopathology [24].

The attractiveness of the human model was significantly different across subgroups, and BIW and BIM chose a figure with a higher BMI than other subgroups. Women with EDs chose thinner human models than those without EDs, and this finding supports the presence of a possible internalized cultural thinness standard hypothesis as a maintaining factor in EDs [13]. In addition, for the ideal male sizes, attractiveness was the only factor that exhibited significant differences. The male sexual minorities were more likely than HEM to prefer higher BMI bodies, confirming the social drive to achieve muscularity/higher weight [71]. Furthermore, BIW and HEW chose heavier models than lesbian women, demonstrating that sexual attractiveness is a significant factor that has to be taken seriously by both women and men.

The hypothesis motivating our second research goal concerns the role of sexual orientation in the body image of people with an ED. This is a neglected topic in the literature, particularly its relevance for women. Our data are consistent with the idea that there is a relationship between sexual orientation and body dissatisfaction in non-clinical women, but sexual orientation plays no specific role in body image when a woman has an ED [8, 72, 73]. Indeed, no significant differences in perceptual distortion and body weight dissatisfaction were found between subgroups. Women with an ED had higher psychopathology levels than non-clinical subgroups, which is in line with recent reviews of the literature [24, 74]. However, we observed no differences in body concerns between different sexual orientations. A potential explanation could be that our data do not take masculinity/femininity into consideration, which is influences body dissatisfaction in sexual minority women [75], and sexuality, which studies have shown to be highly correlated with body dissatisfaction [76].

Our third hypothesis was about the relationship between physical comparison, self-esteem, eating concerns, and body weight dissatisfaction across the sexual orientation spectrum. Our observations show that body weight dissatisfaction was most common among BIW and gay men. As suggested by previous studies, the cultural background could significantly impact body weight dissatisfaction [77], but the instrument used could also make a difference. Indeed, a study using silhouettes found the persistence of a discrepancy between the actual silhouette and the one chosen by the participants [78]. In the subgroup correlations analysis, this relationship was not valid for BIW and gay men; for these groups, there was no relationship between these factors. These results are in line with previous research that showed how gay men are exposed to body weight dissatisfaction, regardless of age. The results also demonstrate that BIW have to be considered as a vulnerable group; this group has been understudied [24, 25, 79, 80]. The mediation analyses demonstrated how important the comparison of physical appearance is. The physical appearance comparison directly and indirectly affects body weight dissatisfaction in women and a mediator role for the body misperception across all the cisgender population. Moreover, these data confirmed how meaningful physical comparison is for cognitive body bias and body dissatisfaction [81–83]. Thus, physical comparisons should be considered as an essential target for the reduction of body misperception and the reduction of body weight dissatisfaction in women. Indeed, attentional bias studies have demonstrated that there may be a judgment and memory bias about body shapes and satisfaction [20], with a possible role of cognitive biases in body weight dissatisfaction. More studies are needed to identify effective methods that can change the way physical comparison influences body dissatisfaction, or how cognitive bias can be improved [84–86]. For example, the “exposure with response prevention” is a cognitive-behavioral technique that has already demonstrated preliminary efficacy in modifying body evaluation with targeting interventions on ritualistic behaviors and thoughts due to physical comparison. However, the results are still preliminary [87, 88]. In the male population, the mediation analysis showed that eating concerns has a mediator role between sexual orientation and bodyweight dissatisfaction, corroborating the role of eating concerns in the evaluation of own bodyweight in gay men and showing a possible target for interventions focused on the improvement of their weight dissatisfaction that could bring to unhealthy behaviors [65].

## Limitations

This study has some limitations that must be considered. First, the data were collected from a large sample and with a variety of methods to assess body image. Quantitative analyses have been applied with a possible reduction of salient aspects that could be evaluated. Unfortunately, we could not recruit enough sexual minority men with an ED to allow for sound statistical analyses. Second, this study was not able to compare all sexual orientations in men and women; doing so could enable us to infer relationships among different subgroups. Third, while online surveys have demonstrated sufficient reliability [89], they rely on collecting self-reported responses, which should be viewed with caution. Fourth, the decision to include only cisgender responders has reduced the relationship between sexual orientation and their own body. Still, this decision helps simplify the analysis of the results regarding the role of sexual orientations. Finally, the exclusive inclusion of participants who have a computer and internet access should be considered a limitation. Therefore, this study’s results should be considered exploratory, but they advocated for more research in the relationship between sexual orientation, body weight dissatisfaction, and ED psychopathology.

## Conclusion

Our data support the hypothesis that gender (female/male) and sexual orientations have an influence on body weight dissatisfaction and body perception/representation in the cisgender population. This study found significant relationships between the sexual orientations of cisgender individuals and body weight dissatisfaction and body size perception. Bisexual women and gay men seem to be the most vulnerable to body weight dissatisfaction, which is a significant risk factor for psychiatric disorders with severe impacts on patients’ lives. However, the data collected should be increase with more studies with integrated methodology (i.e., with qualitative data) that allowed a deeper inside into the effect of body weight dissatisfaction or body perception. For this reason, sexual orientation should be systematically taken into consideration in the assessment and treatment of EDs or other body image disturbances (e.g., dysmorphophobic disorder).

## What is already known on this subject?

The relationship between sexual orientation and body weight dissatisfaction and body representation is a neglected topic, especially in women and sexual minorities. It has a role in the development and in the maintenance of body image disturbance, but there is a lack of research about the presence of any moderator constructs that could contribute to its modification.

## What does this study add?

A large sample of cisgender women and men was included in the study, showing how that bisexual women and gay men are exposed to higher body weight dissatisfaction and distorted body shapes perception. Attractiveness analysis highlighted the relevance of internalized normative pressure for thinner female bodies in all subgroups, even though bisexual women and men prefer healthy weight bodies. Physical appearance comparison is a crucial psychological element in the assessment of body comparison and evaluation, and this should be taken into consideration in mental health prevention programs.

## Data Availability

The datasets generated and analyzed during the current study are available from the corresponding author on reasonable request.
